# Endometrial cancer cells exhibit high expression of p110β and its selective inhibition induces variable responses on PI3K signaling, cell survival and proliferation

**DOI:** 10.18632/oncotarget.13989

**Published:** 2016-12-16

**Authors:** Thomas Karlsson, Camilla Krakstad, Ingvild Løberg Tangen, Erling A. Hoivik, Pamela M. Pollock, Helga B. Salvesen, Aurélia E. Lewis

**Affiliations:** ^1^ Department of Molecular Biology, University of Bergen, Bergen, Norway; ^2^ Centre for Cancer Biomarkers, Department of Clinical Science, University of Bergen, Bergen, Norway; ^3^ Department of Biomedicine, University of Bergen, Bergen, Norway; ^4^ Department of Gynecology and Obstetrics, Haukeland University Hospital, Bergen, Norway; ^5^ Queensland University of Technology, Brisbane, Australia

**Keywords:** PIK3CB, p110β, PTEN, endometrial cancer, PI3K inhibitors

## Abstract

PTEN loss and constitutive activation of the class I phosphoinositide 3-kinase (PI3K) pathway are key drivers of endometrial tumorigenesis. In some cancer types, PTEN-deficient tumors are reliant on class I PI3K p110β (encoded by *PIK3CB*) activity but little is known about this contribution in endometrial tumorigenesis. In this study, we find that p110β is overexpressed in a panel of 7 endometrial cancer cell lines compared to non-transformed cells. Furthermore, in 234 clinically annotated patient samples, *PIK3CB* mRNA levels increase significantly in the early phase of tumorigenesis from precursors to low grade primary malignant lesions whereas *PIK3CA* levels are higher in non-endometrioid compared to endometrioid primary tumors. While high levels of either *PIK3CA* or *PIK3CB* associate with poor prognosis, only elevated *PIK3CB* mRNA levels correlate with a high cell cycle signature score in clinical samples. In cancer cell lines, p110α inhibition reduces cell viability by inducing cell death in *PIK3CA* mutant cells while p110β inhibition delayed proliferation in PTEN-deficient cells, but not in WT cells. Taken together, our findings suggest that *PIK3CB*/p110β contributes to some of the pleiotropic functions of PI3K in endometrial cancer, particularly in the early steps by contributing to cell proliferation.

## INTRODUCTION

The class I phosphoinositide 3-kinase (PI3K) pathway is frequently altered in cancer via genetic alterations in several gene pathway members, contributing to uncontrolled cell proliferation and survival [1, 2]. Class IA consists of heterodimers of catalytic subunits (p110α, p110β and p110δ encoded by *PIK3CA*, *PIK3CB* and PIK3C*D* respectively) and adaptor proteins (p85α and p85β encoded by *PIK3R1* and *PIK3R2* respectively) [3] which phosphorylate phosphatidylinositol (4,5)-bisphosphate (PtdIns(4,5)*P*_2_) to phosphatidylinositol (3,4,5)-trisphosphate (PtdIns(3,4,5)*P*_3_). This reaction is opposed by PTEN (phosphatase and tensin homolog), which de-phosphorylates PtdIns(3,4,5)*P*_3_ to PtdIns(4,5)*P*_2_, hence limiting the effects of class I PI3K activity [4]. In cancer, activating *PIK3CA* mutations are frequent [5, 6] while *PIK3CB* is mutated less often. So far, two activating mutations in *PIK3CB* have been characterized [7, 8], one of which has been discovered in a few cancer types by whole exome sequencing [8]. p110β can however promote oncogenic transformation when over-expressed [9] and has also been shown to be the key isoform mediating tumorigenesis in PTEN-deficient breast and prostate cancers [10–14]. A study by Juric *et al* further highlighted the importance of p110β in tumorigenesis [15] by showing that *PIK3CA* mutant tumors, which were initially sensitive to p110α inhibition, eventually developed resistance due to acquired loss of PTEN. Resistance to p110α inhibition could however be overcome when treatment with a p110β inhibitor was introduced. Other studies have however shown that certain tissues with PTEN loss become dependent on p110α rather than p110β [16]. These contrasting studies indicate the importance of studying isoform-dependence associated with PTEN-loss in each tissue as this may have significant therapeutic implications.

Endometrial cancer is the most common gynecological malignancy in developed countries. Endometrial tumors have been traditionally divided into two groups, type I and type II, according to clinical, pathologic and molecular features. About 80% of diagnosed cases are comprised of the histologic subtype endometrioid endometrial cancer (EEC) and are classified as type I. These tumors are more often estrogen-dependent, linked to obesity, low grade and stage and with good prognosis if treated early. On the other hand, type II, or non-endometrioid endometrial cancer (NEEC), are usually estrogen-independent with serous, clear cell or undifferentiated morphology, high grade and stage and with poor prognosis. Recent whole exome sequencing and integrative genomic profiling led to a molecular-based sub-classification of EEC and NEEC tumors [17–19]. The PI3K pathway is the most frequently altered pathway in EEC with more than 80% of tumors harboring somatic alterations in at least one gene member of the pathway, including high frequency mutations in *PTEN*, *PIK3CA* and *PIK3R1* and low frequency in *AKT* and *PIK3R2* [20–22]. Loss-of-function mutation of the tumor suppressor gene *PTEN* is the most common genetic event in EEC and occurs as an early event in 18-50% of lesions with atypical hyperplasia [23–25]. *PIK3CA* is frequently mutated in 10-39% of EEC but in contrast to *PTEN* has a higher frequency in high grade, aggressive, invasive and less differentiated tumors [24, 26, 27]. *PIK3CA* gene amplification can also account for other mechanisms for PI3K pathway activation and was found to correlate with a PI3K activation profile which segregated more frequently to a group of aggressive and invasive tumors, notably in NEECs. In contrast to *PIK3CA*, mutation events are rare in *PIK3CB* with 2.3% in endometrial cancer according to data from COSMIC (release v72 http://cancer.sanger.ac.uk/cosmic [28], including a recently characterized oncogenic mutation in its catalytic domain [8]). *PIK3CB* mRNA levels were found to be elevated in endometrial tumors compared to normal tissue in a few patient samples [29]. Overexpression of the p110β isoform is thus a possible explanation for the oncogenic properties of the wild type form of this isoform, but this is largely unknown particularly in endometrial cancer. Considering that PTEN loss and PI3K pathway activation are known key drivers of carcinogenesis in endometrial cancer, we hypothesized that p110β could play a significant role particularly in PTEN-deficient tumors. We therefore explored the cellular function and signaling properties of p110β compared to those of p110α in a panel of PTEN-positive and PTEN-deficient endometrial carcinoma cell lines. Finding that the protein levels of p110β, but not p110α, were upregulated in most endometrial carcinoma cell lines, we then demonstrated the distinct contribution of p110α and p110β to cell survival, proliferation and signaling depending on the presence of *PTEN* and *PIK3CA* mutations, using selective pharmacological inhibitors. Furthermore, the potential clinical relevance for these findings were substantiated by exploring an extensively clinically annotated patient cohort with 234 samples ranging from precursors through different stages of dedifferentiation during tumorigenesis demonstrating an increase in *PIK3CB* mRNA levels in early endometrioid lesions that associated with a high cell cycle progression score and decreased survival.

## RESULTS

### p110β levels are elevated in endometrial cancer cell lines and increase from precursors to invasive lesions in clinical samples correlating with reduced survival

**Figure 1:**
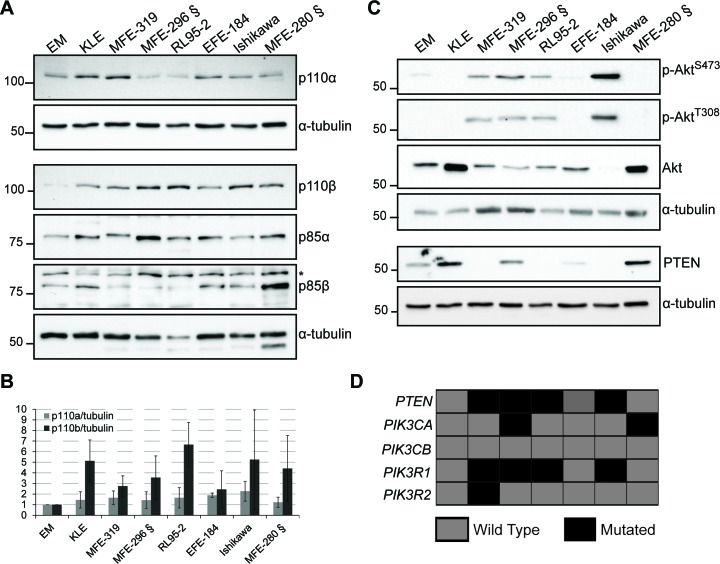
p110β levels are elevated in endometrial cancer cell lines **A**. and **C**. Whole cell extracts obtained from actively growing cells analyzed by Western immunoblotting with the indicated antibodies. α-tubulin was used as a loading control. § indicate *PIK3CA* mutant cell lines. * indicate a non-specific band. **B**. p110β/α-tubulin and p110α/α-tubulin ratios shown as fold increase compared to EM cells from 3-4 independent experiments with standard deviations. **D**. Chart showing the mutational status of each gene and for each cell line (gray, WT; black, mutated gene).

**Figure 2:**
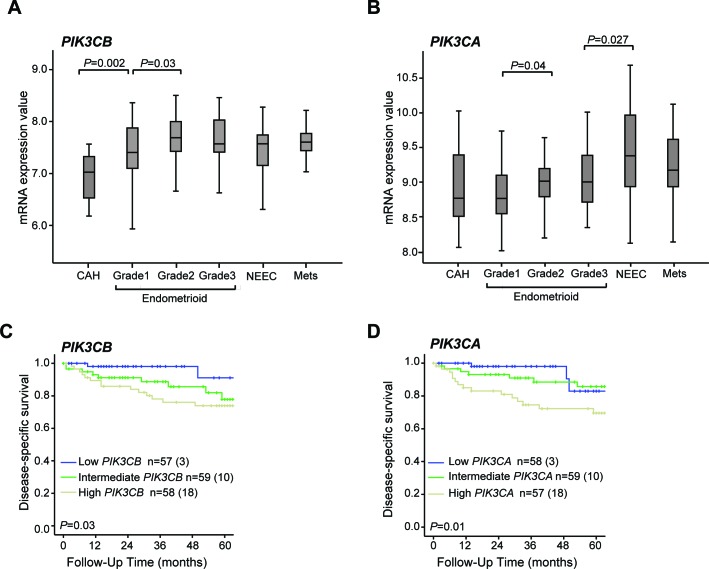
*PIK3CB* mRNA levels increase from precursors to low grade endometrial carcinoma lesions which associates with lower survival Box-plots showing *PIK3CB* **A**. and *PIK3CA* **B**. mRNA levels in relation to histological type and grade. Significant Kruskall Wallis test p-values are given above the relevant box plots. CAH: Complex atypical hyperplasia (*n* = 18). Endometrioid grade 1 (*n* = 48), grade 2 (*n* = 53), grade 3 (*n* = 39). NEEC: Non endometrioid endometrial carcinoma (*n* = 34). Mets: Metastatic lesions (*n* = 42). Kaplan-Meier survival curves shown for patients with high, medium and low mRNA levels according to tertile limits for *PIK3CB* **C**. and *PIK3CA* **D**. Numbers in brackets represent number of disease specific deaths in each group.

We have examined the protein levels of the class I PI3K catalytic (p110α and p110β) and regulatory subunits (p85α and p85β), PTEN, Akt and phosphorylated Akt on serine 473 (p-S473-Akt) and threonine 308 (p-T308-Akt) in whole cell extracts of 7 endometrial cancer cell lines versus a non-tumor immortalized endometrial cell line (EM). In actively growing cells, the levels of p110β were elevated in endometrial cancer cell lines compared to EM cells, independently of their mutational status, whereas the levels of p110α demonstrated less change (Figure 1A-1B). The levels of p85α in the cancer cells were comparable to EM cells but lower levels of p85β were observed in several of the cancer cell lines. To determine the PI3K signaling status, we examined the levels of p-S473-Akt and p-T308-Akt versus total Akt in actively growing cells (Figure 1C). Consistent with other studies [30], all cell lines with PTEN mutations (MFE-296) and exhibiting protein loss (MFE-319, RL-95-2 and Ishikawa) displayed high p-Akt levels (Figure 1C-1D – *c.f*. quantifications in Supplementary Figure S1). Interestingly, MFE-280 cells, which express PTEN but harbor mutations in *PIK3CA*, demonstrated low levels of p-S473/T308-Akt (Figure 1C-1D), consistent with the notion that PTEN loss and *PIK3CA* mutation can have different effects on Akt activation. Low p-Akt levels have previously been observed in breast cancer cell lines with *PIK3CA* mutations, demonstrating possible Akt-independent effects of some mutations in *PIK3CA* [31–33]. We also analyzed the mRNA levels of *PIK3CB* compared to *PIK3CA* in a cohort of endometrial cancer patients, including 18 with complex atypical hyperplasia (CAH), 174 primary tumors and 42 metastatic lesions. *PIK3CB* mRNA levels were significantly increased from precursor lesions with CAH to grade 1 EEC lesions and remained constant in higher grades, NEEC and metastatic tumors (Figure 2A). These results suggest that increased levels of *PIK3CA*/p110β contribute to the early phase of endometrial tumorigenesis. In contrast, a distinct pattern was seen for *PIK3CA* mRNA levels being significantly elevated in NEEC (Figure 2B). Furthermore, elevated levels of both *PIK3CA* and *PIK3CB* were also reflected in lower disease specific survival (Figure 2C-2D).

### *PIK3CA* mutant endometrial cancer cells respond to p110α inhibition by a decrease in cell survival

**Figure 3:**
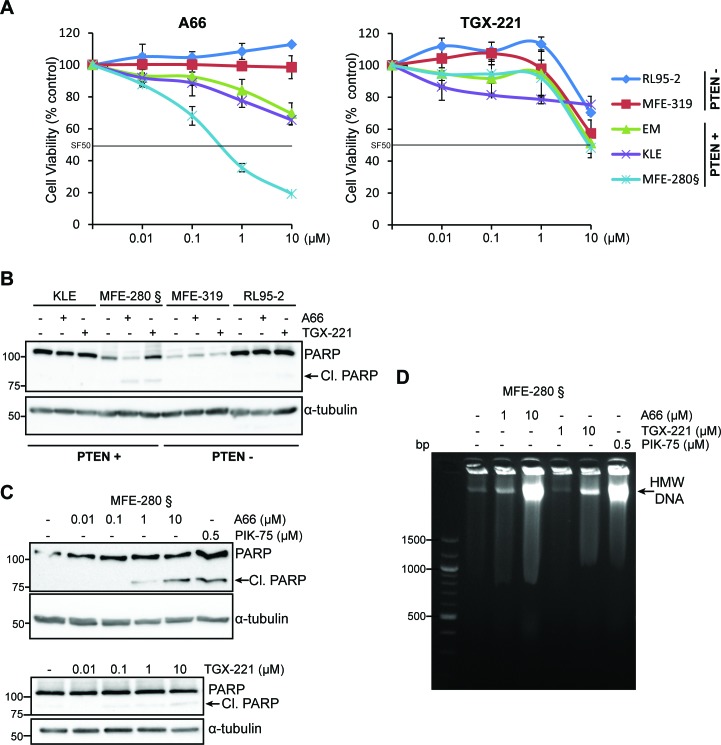
*PIK3CA* mutation and PTEN expression status influence differently the sensitivity of p110α and p110β inhibitors to cell survival in endometrial carcinoma cells **A**. Cell viability determined by MTS assay in PTEN-positive (PTEN+) (EM, KLE and MFE280) and PTEN-deficient (PTEN-) cell lines (RL95-2 and MFE-319) of which only MFE-280 harbor mutated *PIK3C*A (§), treated with 0.01-10 μM of A66 or TGX-221 for 72 h. Data is presented for each cell line as a % relative to DMSO control (mean of 3 independent experiments in triplicates + SDs). Surviving cell fraction of 50% (SF50) threshold is indicated as a black line. **B**. Whole cell extracts were obtained from cells treated with 10 μM of the p110α inhibitor A66 or p110β inhibitor TGX-221 for 24 h and analyzed by Western immunoblotting for PARP cleavage and α-tubulin levels. **C**. Whole cell extracts were obtained from MFE-280 cell treated with 0.01-10 μM of A66 or TGX-221 and 0.5 μM PIK-75 for 24 h and analyzed by Western immunoblotting for PARP cleavage and α-tubulin levels. **D**. Agarose electrophoresis of high molecular weight DNA fragmentation of MFE-280 floating cells collected following treatment with 1-10 μM of A66 or TGX-221 and 0.5 μM PIK-75 for 24 h.

PTEN-deficient cancer cells have been suggested to rely upon p110β signaling for their sustained cell proliferation and tumorigenesis [10, 13]. To investigate whether endometrial cancer cell functions are also influenced by PTEN expression status and if they are dependent upon p110α and/or p110β, we evaluated the cell viability of PTEN-positive (EM, KLE and MFE-280) and PTEN-deficient cell lines (MFE-319 and RL95-2) following treatment with the selective p110α (A66) and p110β (TGX-221) inhibitors (Figure 3A) [34]. Inhibition of p110α had no effect in PTEN-deficient cells and had only a marginal effect in KLE and EM cells with WT PTEN. In contrast, a dose-dependent decrease in cell viability was induced in MFE-280 cells harboring an activating mutation in *PIK3CA* (H1047Y), reaching an SF_50_ (50% surviving cell fraction) at < 1 μM. All cell lines had little response to inhibition of p110β with TGX-221 and required ≥ 10 μM to reach an SF_50_. To investigate if the observed decrease in cell viability in MFE-280 cells was due to an induction of apoptosis, cells were analyzed by Western immunoblotting for poly(ADP-ribose) polymerase 1 (PARP) cleavage following treatment with A66 and TGX-221 (Figure 3B-3C). Incubation with A66 and TGX-221 had no effect in KLE, MFE-319 or RL95-2 cells on PARP cleavage (Figure 3B and Supplementary Figure S2A-B). In contrast, PARP cleavage was induced by A66 in a dose-dependent manner in MFE-280 cells as cleavage was apparent at 1 μM and more strongly at 10 μM (Figure 3B-3C). Inhibition of p110β with TGX-221 had a much weaker effect on PARP cleavage in these cells (Figure 3B-3C). Interestingly, A66 and TGX-221 induced similar effects on the production of high molecular weight DNA fragments but without the generation of oligosomal DNA fragments (Figure 3D), perhaps indicative of the induction of an alternative type of cell death as previously observed in other studies [35–37]. These results suggest that p110α inhibition preferentially triggers a decrease in cell viability and induces cell death in *PIK3CA* mutant MFE-280 cells, and not in *PIK3CA* WT KLE cells. PTEN-deficient cells were in contrast resistant to both p110α and p110β inhibition.

### p110β inhibition impairs cell proliferation in PTEN-deficient cells but with variable responses

**Figure 4:**
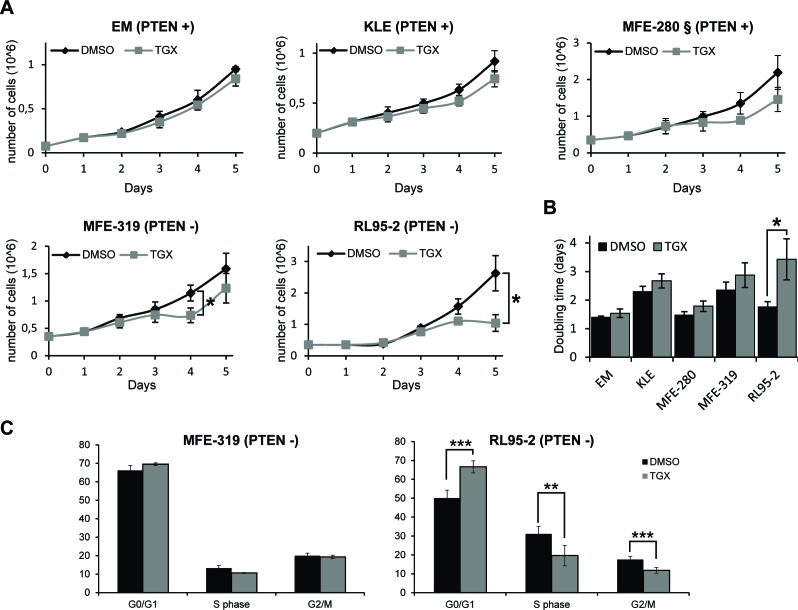
Inhibition of p110β delays cell proliferation in PTEN-deficient endometrial cancer cells **A**. Cell lines were plated at day 0 and treated with DMSO (black line) or with 10 μM TGX-221 (grey line) at day 1 and day 3 and the number of cells were counted at all the indicated days. Cell numbers are presented as means of 3 independent experiments in triplicates for each day + SDs, t-test, * *P* < 0.05. The *PIK3CA* mutant cell line MFE-280 is marked with §. **B**. Doubling time calculated from data acquired in (A) for each cell line. **C**. MFE-319 and RL95-2 cells were treated with DMSO or 10 μM TGX-221 for 24 h and the percentage of cells in each phase of the cell cycle was determined by flow cytometry. Data are means +/− SDs of 3-4 independent experiments and Student's *t* tests were performed comparing TGX-221-treated cells to DMSO-treated cells for each cell cycle phase with ** *P* < 0.01 and *** *P* < 0.001.

p110β has previously been shown to regulate cell proliferation [10, 38] particularly in PTEN-deficient tumors [13]. Since p110β inhibition did not induce PARP cleavage in PTEN-deficient cells, we tested if an effect could be apparent on cell proliferation, using the highest concentration of TGX-221 (10 μM) which showed a 50% decrease in cell viability in some cell lines (Figure 3A). While no significant decrease in cell number was observed for the PTEN-positive cell lines, p110β inhibition induced a significant decrease in the number of cells in the PTEN-deficient cell lines RL95-2 and MFE-319 following 4 and 3 days of treatment respectively (Figure 4A). However, after 4 days of treatment, the cell division rate of MFE-319 cells returned to that of control cells. This was reflected by a significant increase in total doubling time in RL95-2 but not in MFE-319 cells following TGX-221 treatment (Figure 4B). p110β has also been shown to contribute to G1 to S phase progression [39, 40] and we therefore evaluated if p110β inhibition affected the cell cycle distribution of the PTEN-deficient cells. As shown in Figure 4C, RL95-2, but not MFE-319 cells, demonstrated an increase in the percentage of cells in G1 phase and a concomitant decrease in the percentage of cells in S and G2/M phases compared to control cells. These results demonstrate that p110β can contribute to cell proliferation by regulating G1 to S phase progression to a certain extent but only in a subset of PTEN-deficient cells.

### High *PIK3CB* mRNA levels correlate with a high cell cycle progression gene signature score in clinical samples

**Figure 5:**
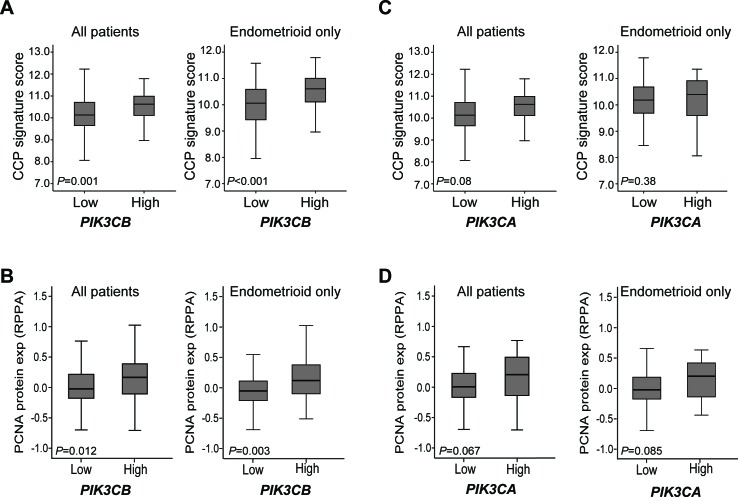
High *PIK3CB* mRNA levels associates with high cell proliferation markers in primary tumors Box-plots are shown for the cell cycle progression (CCP) signature score in relation to low versus high *PIK3CB* **A**. or *PIK3CA* mRNA levels **C**. and for PCNA protein levels in relation to low versus high *PIK3CB* **B**. or *PIK3CA* mRNA levels **D**., either in all primary tumors or endometrioid subgroup tumors.

To further explore the clinical relevance of the observed contribution of p110β activity in cell proliferation in cell line studies, we analyzed potential phenotypic links between mRNA levels of both *PIK3CB* and *PIK3CA* in clinically annotated endometrial carcinoma samples with a cell cycle progression (CCP) score established from 31 CC genes [41]. High *PIK3CB* mRNA levels were significantly associated with high level of CCP signature score and high protein levels of the proliferation cell nuclear antigen (PCNA) for all primary tumors and notably within the endometrioid subgroup (Figure 5A-5B). In contrast, *PIK3CA* mRNA levels did not associate with measures for cell proliferation when explored for all primary tumors or the endometrioid subgroup (Figure 5C-5D). These results suggest that *PIK3CB*/p110β contributes to endometrial tumorigenesis by influencing the cell cycle regulation of endometrial cancer cells. In contrast, poor disease specific survival observed with high *PIK3CA* levels (Figure 2D) is more likely due to other PI3K-mediated cellular processes than proliferation.

### PTEN positive and deficient endometrial cancer cells respond differently to p110α and p110β inhibition on Akt and S6 signaling

**Figure 6:**
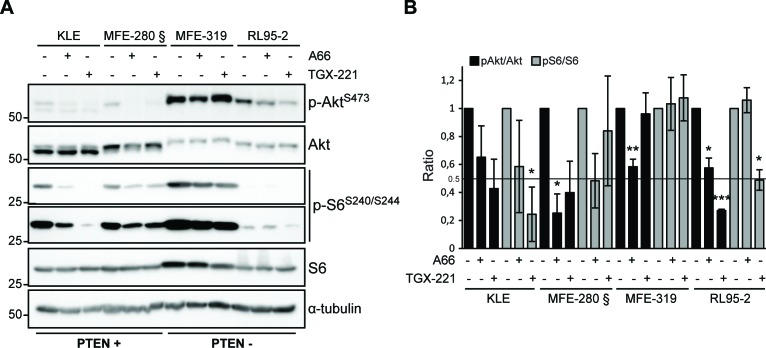
Heterogeneous response on Akt and S6 signaling following p110α and p110β inhibition **A**. Whole cell extracts were obtained from cells treated with 10 μM of A66 or TGX-221 for 24 h and analyzed by Western immunoblotting for pS473 Akt, Akt, pS240/244 S6 ribosomal protein (short and long exposures are shown), S6 and α-tubulin levels. **B**. Quantification of pS473-Akt/Akt and pS240/244-S6/S6 ratios relative to DMSO-treated controls for each cell line from at least 3 independent experiments. Standard deviations and significance level for difference (T-test compared to DMSO-treated cells: *P < 0.05, **P < 0.01, ***P < 0.001) are shown for each condition.

To correlate cellular function with PI3K pathway signaling, we determined the effect of both p110α and p110β inhibitors on AKT (p-S473-Akt) and mTORC1 (p-S240/S244-S6) signaling (Figure 6). All cell lines, whether PTEN-positive with low p-S473-Akt or PTEN-deficient with high p-S473-Akt, responded to p110α inhibition with a decrease in Akt basal activity. Interestingly, the best response to p110α inhibition, measured both by decreased p-Akt and cell viability, was observed in the *PIK3CA* mutant MFE-280 cells. Inhibition of p110β also led to a decrease in p-S473-Akt in all cells except the PTEN-deficient cell line MFE-319. Response to p110α inhibition on S6 signaling was varied from a low response in PTEN-positive cells to no response at all in PTEN-negative cells. Response to p110β inhibition on S6 signaling was even more varied and independent of PTEN expression status. The PTEN-deficient RL95-2 cells were responsive while MFE-319 cells were resistant. The PTEN-positive cells KLE were very responsive while MFE-280 cells were resistant.

## DISCUSSION

All class I PI3K isoforms can produce PtdIns(3,4,5)*P*_3_, the levels of which are controlled by at least the action of PTEN. In lesions with loss of *PTEN*, tumorigenicity is thought to rely on the activity of not only p110β but also of p110α depending on the tissue type [10, 13, 16, 42]. In EEC, *PTEN* is the most commonly mutated gene [43] but little is known however about the exact contribution of p110β in endometrial tumor development particularly in relation to *PTEN* loss. In this study, we demonstrate that p110β is highly expressed in endometrial cancer cell lines and that its mRNA levels increase in clinical samples during the initial steps of endometrial carcinogenesis for the endometrioid subtype. In contrast, high *PIK3CA* mRNA levels were observed in non-endometrioid tumors, which would appear to be consistent with previous findings linking *PIK3CA* gene amplification to aggressive endometrial tumors of non-endometrioid histology [44]. In addition, different roles were found for the two isoforms in this study, as supported by the association of high mRNA levels of *PIK3CB*, but not *PIK3CA*, with markers for high proliferation (CCP score and PCNA levels), notably in endometrioid tumors. Interestingly, the CCP score was reported to increase early in grades 1 and 2 compared to CAHs [45], at the same stage of tumor development when *PIK3CB* mRNA levels are found to be increased in this study. Hence, high *PIK3CB* mRNA levels may provide a proliferative advantage in early stages of tumorigenesis. Using pharmacological inhibition of p110β, our cell line studies also pointed to a role for p110β in cell proliferation since its inhibition delayed cell proliferation by arresting cells in G1 in a PTEN-deficient cancer cell line, RL95-2. Consistently, p110β has previously been shown to be localized in the nucleus [40, 46, 47] and to mediate S phase entry, control DNA replication and loading of PCNA on chromatin [40]. In functional studies of nuclear p110β [40, 48], TGX-221 was previously used at high concentration (30 μM), which was shown to be specific for p110β and not p110α [40]. In this study, inhibition of proliferation was only apparent with 10 μM of the inhibitor, which may be due to high levels of p110β in endometrial cancer cell lines as well as its subcellular localization spanning not only the cytoplasm but also the nucleus in other cell lines [46, 47] and the endometrial cancer cells used in this study (data not shown). Although we cannot exclude off-target effects at 10 μM, an extensive study of TGX-221 at that concentration on a large panel of lipid and protein kinases by Jamieson *et al* showed high specificity for p110β [34].

The reported association between p110β level and activity with cell proliferation suggests further clinical testing of endometrial tumors with *PTEN* mutations for potential benefit from p110β selective inhibitors to delay tumor growth. This is also particularly relevant in light of a recent study showing response to inhibition of p110β in PTEN-deficient breast cancer cells, resistant to p110α inhibition [15]. However, we observed that PTEN-deficient cancer cells can respond with different efficacy following p110β inhibition (MFE-319 versus RL95-2 cells) and resistance to treatment was observed in MFE-319 cells (Figure 4). This would be consistent with a recent study by Schwartz *et al* showing that p110α was reactivated following p110β selective inhibition in PTEN-deficient cells [49]. The efficacy of p110β inhibition may hence have limited durability and combined or sequential treatment targeting p110β and p110α may be necessary in tumors with PTEN loss. Other genetic factors may also influence the efficacy of p110β inhibition, such as the presence of *RAS* mutations, since the RAS-MEK and PI3K pathways are known to converge [50]. Indeed RL95-2 cells harbor an activating mutation in *H-Ras* (Q61H), which may explain the necessity of high concentration of TGX-221. PTEN-deficient cells with RAS mutation may benefit from a combination of p110β and RAS/MEK pathway inhibition. The cause for resistance to p110β inhibition in MFE-319 cells is not yet known but it is worth noticing that these cells harbor a mutation in the fibroblast growth factor receptor 2 (S252W) shown previously to be oncogenic and to occur with high frequency in endometrial cancer [51, 52]. However, although FGFR signaling is known to activate the PI3K pathway, MFE-319 cells were shown to be insensitive to FGFR inhibition [53]. In any case, identifying the molecular mechanisms of resistance to p110β inhibition in a subset of PTEN-deficient tumors is crucial to design clinical therapeutic strategies with long-term efficacy. In addition, this would anticipate understanding of the results of a phase I/IIa clinical trial with a selective p110β inhibitor in carcinomas with PTEN loss, including endometrial cancer (NCT01458067) and which has recently been completed [54].

The PTEN-positive MFE-280 cells harboring a *PIK3CA* mutation in its catalytic domain (H1047Y) responded to selective p110α inhibition with a decrease in cell viability and an increase in cell death while *PIK3CA* WT and PTEN-deficient cell lines were resistant. The observation that these cells were more sensitive to p110α inhibition than WT *PIK3CA* is in agreement with the studies of Torbett *et al* [12] showing that breast cancer cells with *PIK3CA* mutations in their catalytic domain responded to the p110α inhibitor PIK-75, but not PTEN-deficient cells. Consistently, T47D breast cancer cells with the H1047R mutation in *PIK3CA* were sensitive to the p110α inhibitor BYL719 measured by cell viability assay, but exhibited a lower response when PTEN was knocked down [15]. We also showed that inhibition of p110β had little effect on cell viability and required >10 μM TGX-221 to reach 50% cell survival in most endometrial cancer cell lines studied, including PTEN-deficient cells. This is in agreement with the work of Weigelt *et al* [30] in endometrial cancer cells but in contrast to studies in breast and prostate cancer [10, 11, 13], arguing that p110β does not contribute to cell survival in endometrial cancer cells. In this study, we showed that the *PIK3CA* mutant cell line MFE-280 reached an SF^50^ of 10 μM in cell viability assay and was the only cancer cell line where TGX-221 induced cell death, albeit weakly, and only at the highest dose at 10 μM, hence with low efficacy. This may suggest an active cooperation between p110α and p110β. A recent study has indeed showed that following serum stimulation, activated p110α associates with a fraction of p110β and leads to p110β activation [55]. Accordingly, the activating mutation in *PIK3CA* found in MFE-280 cells may allow the recruitment of p110β to p110α and its subsequent activation. The resulting activation of both isoforms would hence allow these cells to respond to inhibitors selective for either isoform. The association of p110β with p110α would implicate that both isoforms can engage in similar cellular function, in this case cell survival, and respond to similar external cues. On the other hand, the remaining fraction of p110β not associated with p110α could still engage in other cellular functions including proliferation.

Different effects on PI3K isoform-specific signaling were demonstrated to be PTEN context dependent for p110α and p110β selective inhibition. Decrease in p-Akt was observed with variable efficacy with both p110α and p110β inhibitors, independently of PTEN status and despite their differential effects on cell viability. In contrast, a different response to p110α inhibition was apparent for p-S6 which was lower in PTEN-positive but not in PTEN-deficient cells. This would be consistent with a study in breast cancer cells where persistence of mTORC1 signaling via p-S240/S244-S6 was shown to be a marker of resistance to p110α inhibition [56] although this study did not show any correlation with PTEN status. p110β inhibition decreased p-S6 in RL95-2 cells but not in MFE-319 displaying a more transient effect of p110β inhibition on cell proliferation. The difference in mTORC1/S6 signaling sensitivity to p110β inhibition in PTEN-deficient cells seemed to be correlated with the difference in p-S6 levels, *i.e*. with a higher response in RL95-2 cells that have the lowest p-S6 levels and no response in MFE-319 with the highest levels. In addition, decrease in p-S6 by p110β inhibition correlated with a decrease in cell proliferation in RL95-2 cells. MFE-319 cells may thus have acquired resistance to both p110α and p110β inhibitors via mTORC1 hyper-activation.

In conclusion, our results demonstrate for the first time that high levels of *PIK3CB*/p110β associate with the early stages of endometrial tumorigenesis and increased cell proliferation, suggesting a proliferative advantage. In cell lines, p110β plays a role in a PTEN-dependent context in cell proliferation but not in cell survival. However, the benefits of p110β inhibition may not be lasting and lead to resistance in some PTEN-deficient endometrial tumors. This may be relevant for the design of more effective and combined targeted therapy in future clinical trial in endometrial cancer featuring loss-of-function mutations in *PTEN*.

## MATERIALS AND METHODS

### Reagents

Antibodies used in Western immunoblotting are the following: anti-Akt (2920), -S473-Akt (9271), -pT308-Akt (C31E5E), -p110α (4249), -p110β (3011), -PARP (2730), -S6 (2217) and -pS240/S244-S6 (5364) from Cell Signaling; anti-p85α (05-212) from Millipore; anti-p85β (S3089) from Epitomics; anti-PTEN (7974) from Santa Cruz and anti-α-tubulin (T5168) from Sigma-Aldrich. Horse radish peroxidase-conjugated secondary antibodies were from Life Technologies. The PI3K selective inhibitors targeting p110α were from Selleck Chemicals (A66) and Millipore (PIK-75) and targeting p110β (TGX-221) from Cayman chemicals [34, 57].

### Cell lines and cell culture conditions

Seven endometrial cancer cell lines were chosen to represent different status for PI3K/Akt pathway activation and PTEN protein expression, as shown in this study and by others [21, 30, 43, 58]. Cancer cell lines were obtained from ATCC (KLE, RL95-2), DSMZ Germany (MFE-296, MFE-319, EFE-184 and MFE-280) and Sigma-Aldrich (Ishikawa). EM-E6/E7-hTERT (EM), a non-transformed endometrial cell line isolated from glandular endometrial tissue and immortalized with E6/E7 and human TERT [59, 60], was a gift from Prof PM Pollock (University of Queensland, Australia). All cancer cells were authenticated by short tandem repeat (STR) DNA profiling (IdentiCell Service, Dept. Molecular Medicine, Aarhus University Hospital, Denmark for all cancer cell lines). EM cells were confirmed to have a unique STR profile as shown in Table 1 (MD Anderson Cancer Center, USA). All cancer cells were cultured in Dulbecco's modified Eagle's medium (DMEM) supplemented with 10% fetal bovine serum (FBS) and antibiotics (100 IU/ml penicillin and 100 μg/ml streptomycin). EM cells were cultured in DMEM/Ham's F12 supplemented with Insulin-Transferrin-Selenium, 10% FBS and antibiotics and changed to DMEM containing 10% FBS and antibiotics 24 h before harvest. Cells were harvested when they reached a maximum of 80% confluence.

**Table 1:** STR profile of EM-E6/E7-hTERT cells

Marker	Allele(s)
AMEL	X
CSF1PO	11,12
D13S317	8,9
D16S539	10,11
D18S51	15
D21S11	31.2,32
D3S1358	15,17
D5S818	12,13
D7S820	10,12
D8S1179	10,13
FGA	21,23
TH01	6,7
TPOX	8,11
vWA	14

### Cell viability assay, cell counts and flow cytometry

For cell viability, cells were seeded in 96-well plates with 1000 or 3000 cells/well depending on the cell line, grown for 24 h and treated with inhibitors for 72 h. Cells were then assayed with the CellTiter 96 AQ_ueous_ One Solution Cell Proliferation Assay (Promega) by incubation with 20 μL of 3-(4,5-dimethylthiazol-2-yl)-5-(3-carboxymethoxyphenyl)-2-(4-sulfophenyl)-2H-tetrazolium for 1 h and reading at 490 nm. For cell counts, cells were plated in 6-well plates, grown for 24 h, treated and counted following trypsinization at the same time point every day with a BioRad TC10 automated cell counter. For flow cytometry, 2 x10^5^ cells were seeded in 6-well plates, treated for 24 h and processed as described previously [61]. DNA was stained with 100 μg/mL propidium iodide and the cell cycle distribution was analyzed using a BD Biosciences Accuri C6 flow cytometer. For DNA fragmentation assays, cells were plated in 6-well plates, treated for 24 h and the floating cells were collected, lysed in 40 μL DMSO and 40 μL Tris-EDTA pH 7.4 supplemented with 2% SDS, according to Suman *et al* [62] and half was loaded on a 2% agarose gel.

### Whole cell extracts, subcellular fractionation and Western immunoblotting

Whole cell extracts were prepared in radioimmunoprecipitation assay (RIPA) lysis buffer (50 mM Tris pH 8.0, 0.5% deoxycholic acid, 150 mM NaCl, 1% NP-40, 0.1% SDS) supplemented with 5 mM NaF, 2 mM Na_3_VO_4_ and 1x Sigma Protease Inhibitor Cocktail. Subcellular fractionation was carried out according to O’Caroll *et al.* [63] and nuclear pellets were lysed in RIPA. RL95-2 cells required an additional syringing step of the nuclear pellet resuspended in wash buffer to avoid cytoplasmic contamination. Equal amount of proteins were resolved by SDS-PAGE, immunoblotted as described previously [64] and detected by enhanced chemiluminescence using the SuperSignal West Pico Chemiluminescent Substrate (Pierce) and visualized with a BioRad ChemiDocTM Xrs+.

### Patient series

Fresh frozen tissue was collected from patients diagnosed with endometrial cancer at Haukeland University hospital during the period from 2001-2013 and include a total of 234 clinical samples with 18 endometrial cancer precursor lesions (CAH), 174 primary tumors and 42 metastases. Clinical data were collected as described earlier [25, 45]. The patient cohort used for p110β immunohistochemistry is described in detail in Tangen *et al* [45]. This study was conducted in line with Norwegian legislation and international demands for ethical review, approved by the Norwegian Data Inspectorate, Norwegian Social Sciences Data Services and the Western Regional Committee for Medical and Health Research Ethics (NSD15501; REK 052.01). Patients signed an informed consent form.

### Microarray analyses and reverse phase protein array

Fresh frozen tissue collected in parallel from 18 CAH, 174 primary and 42 metastatic lesions was used for RNA extraction as previously reported [25, 45]. The majority of samples had tumor purity above 80% with a threshold inclusion set at >50%. Samples were gross dissected when necessary to achieve this level of tumor purity. mRNA was extracted from fresh frozen tissue using the RNeasy Mini Kit (Qiagen). Samples were hybridized to Agilent Whole Human Genome Microarrays (G4112F), scanned and normalized as previously reported [65]. mRNA values for *PIK3CA* (probe A_23_P92057) and *PIK3CB* (probe A_23_P346969) were extracted from the microarray dataset. For survival analyses using mRNA values, values were grouped in tertiles, according to similarity in survival and considering the size of the subgroups and the number of events in each category. The CCP signature score was calculated as previously described [45] using the gene expression signature of 31 CCP genes reported by Cuzick *et al*. [41]. For CCP analyses, mRNA values were further grouped in high and low, where the two lowest tertiles were defined as low, and the highest tertile was defined as high. Microarray data are publicly available at ArrayExpress (accession number E-MTAB-2532). Reverse phase protein array for PCNA protein levels was performed as previously described [45].

### Statistical analyses

For clinical samples, statistical analyses were performed using the software package SPSS 22 (SPSS Inc, Chicago, IL) and significance was defined as p < 0.05. Correlations between groups were evaluated using the Mann-Whitney U test for continuous variables. Survival analyses were done using the Kaplan-Meier (product-limit) method. Date of primary surgery was set as entry date and time of death due to endometrial cancer as endpoint (disease specific survival). Survival between groups was compared with log-rank test for trend. For cell lines data, Student's unpaired *t* test with unequal variance was used.

## SUPPLEMENTARY FIGURES



## Abbreviations

CAH: complex atypical hyperplasia
CCP: cell cycle progression
EEC: endometrioid endometrial cancer
NEEC: non-endometrioid endometrial cancer
PI3K: phosphoinositide 3-kinase
PTEN: phosphatase and tensin homolog
PARP: poly(ADP-ribose) polymerase 1
PtdIns(3,4,5)*P*_3_: phosphatidylinositol (3,4,5)-trisphosphate
SF: survival fraction
