# Supplementary material for: Endometrial cancer cells exhibit high expression of p110β and its selective inhibition induces variable responses on PI3K signaling, cell survival and proliferation
Source: Oncotarget. 2016 Dec 16;8(3):3881–94. doi: 10.18632/oncotarget.13989 (PMC5354802; doi:10.18632/oncotarget.13989)
Supplement: Supplementary file 1 [file oncotarget-08-3881-s001.pdf]

Endometrial cancer cells exhibit high expression of p110β and its selective inhibition induces variable responses on PI3K signaling, cell survival and proliferation

Supplementary Material

Figure S1

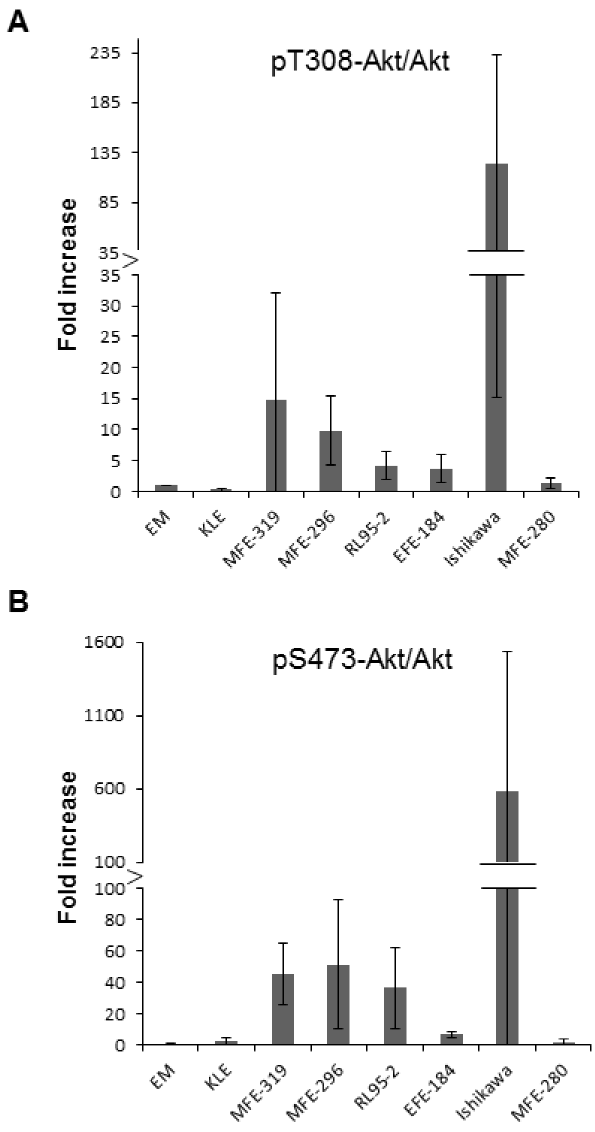

Supplementary Fig. S1: Densitometry of pT308-Akt, pS473-Akt and total Akt signals (related to Figure 1C)
Whole cell extracts obtained from actively growing cells were resolved by Western immunoblotting to detect pT308-Akt, pS473-Akt and total Akt and densitometry of the signals were analysed using Image J. pS473-Akt/Akt and pT308-Akt/Akt ratios were then calculated and shown relative to those obtained in EM cells, for each cell line and from 3 independent experiments ± SDs. > indicates a break in the Y axis.

Figure S2

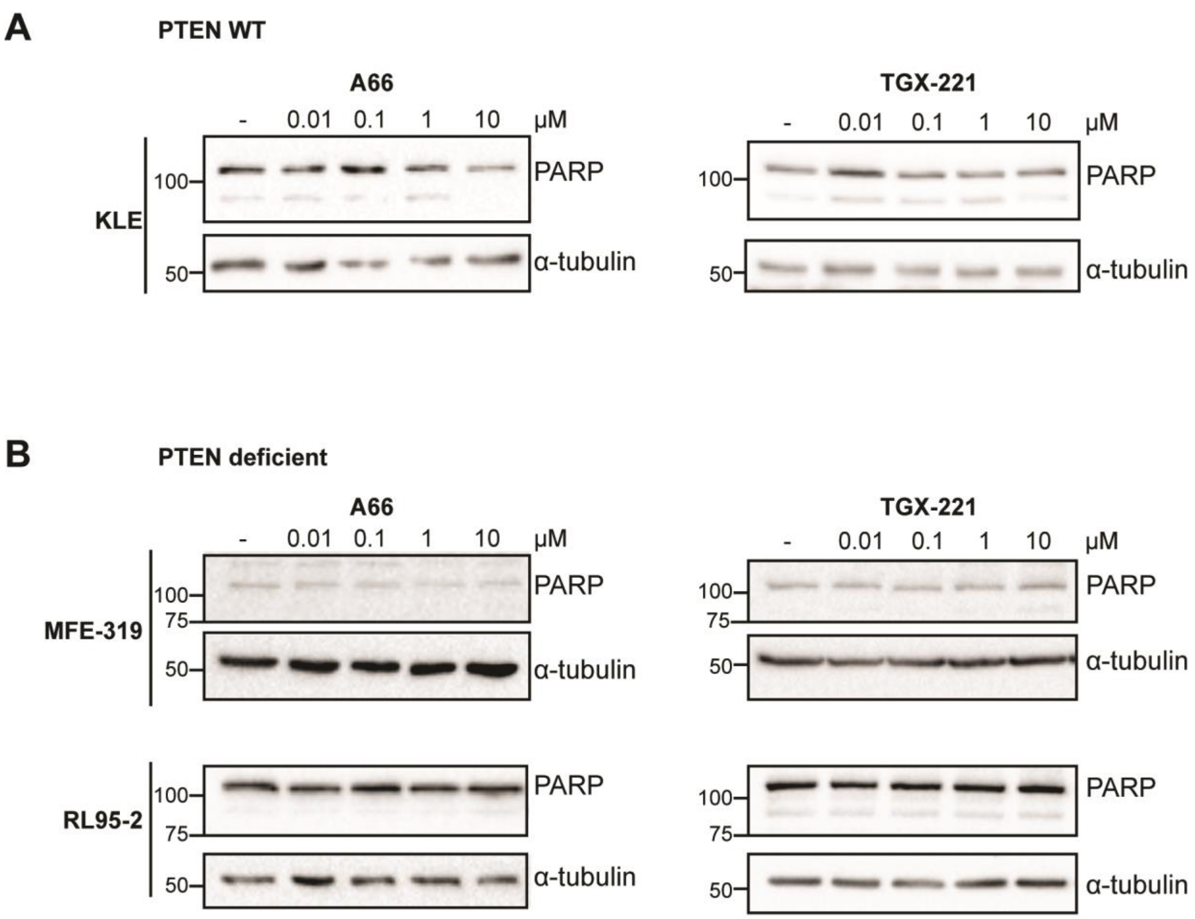

Supplementary Fig S2. Lack of response of EEC cells to p110α and p110β inhibition for PARP cleavage assay

KLE (A), MFE-319 and RL95-2 cells (B) were treated with 0.01-10 μM A66 or TGX-221 for 24h and cell extracts were subjected to Western immunoblotting for poly (ADP-ribose) polymerase (PARP) and α-tubulin.
